# Predictors of rheumatic fever in sore throat patients: a systematic review and meta-analysis

**DOI:** 10.1093/trstmh/trab156

**Published:** 2021-10-12

**Authors:** Ellen Kulik, Beth Stuart, Merlin Willcox

**Affiliations:** School of Primary Care, Population Sciences and Medical Education, Faculty of Medicine, Aldermoor Health Centre, Aldermoor Close, Southampton SO16 5ST, UK; School of Primary Care, Population Sciences and Medical Education, Faculty of Medicine, Aldermoor Health Centre, Aldermoor Close, Southampton SO16 5ST, UK; School of Primary Care, Population Sciences and Medical Education, Faculty of Medicine, Aldermoor Health Centre, Aldermoor Close, Southampton SO16 5ST, UK

**Keywords:** pharyngitis, rheumatic fever, risk factors, streptococcal infections, systematic review

## Abstract

**Background:**

Concerns about rheumatic fever (RF) drive antibiotic prescriptions for sore throat (ST) in endemic areas. Better guidance is needed on which patients are likely to develop RF in order to avoid misuse and overuse of antibiotics. Our aim was to identify predictive factors for RF in ST patients.

**Methods:**

Multiple databases were searched to identify cohort, case–control, cross-sectional or randomised controlled trials that measured RF incidence in ST patients. An inverse variance random effects model was used to pool the data and calculate odds ratios (ORs).

**Results:**

Seven studies with a total of 6890 participants were included: three RCTs and four observational studies. Factors significantly associated with RF development following ST were positive group A streptococcal (GAS) swab (OR 1.74 [95% confidence interval {CI} 1.13 to 2.69]), previous RF history (OR 13.22 [95% CI 4.86 to 35.93]) and a cardiac murmur (OR 3.55 [95% CI 1.81 to 6.94]). Many potential risk factors were not reported in any of the included studies, highlighting important evidence gaps.

**Conclusions:**

ST patients in endemic areas with a positive GAS swab, previous RF history and a cardiac murmur are at increased risk of developing RF. This review identifies vital gaps in our knowledge of factors predicting RF development in ST patients. Further research is needed to develop better clinical prediction tools and rationalise antibiotic use for ST.

## Introduction

Rheumatic fever (RF) is an autoimmune disease triggered by group A streptococcal (GAS) pharyngitis that can affect multiple systems including the joints and cardiac tissue.^1^ It is thought that both genetic and environmental factors play a role in RF susceptibility, although these factors remain virtually unknown.^2–4^

The immune response against the M protein component of GAS leads to the production of autoreactive antibodies and T cells that cross-react with host tissues^4^ such as human cardiac myosin, tropomyosin and laminin,^5^ leading to rheumatic heart disease (RHD).^6–8^ Anti-endothelial cell autoantibodies, which infiltrate the valve surface endothelium, are thought to play a prominent role in cardiac tissue damage in RHD.^8^

RHD is the leading cause of heart failure in children and young adults living in low-income countries.^9^ Much like GAS pharyngitis and RF, RHD onset peaks between the ages of 5 and 15 y.^10,11^ Around 60% of those with RF in endemic communities will subsequently develop RHD.^10^

Both RF and RHD are now less common in developed countries but continue to be seen in indigenous communities and during outbreaks,^12–14^ where RF incidence rates of 38 per 10 000 population have been reported.^15^ In 2015, RHD was estimated to affect 33.4 million people and resulted in 319 400 deaths^16^ through mechanisms such as heart failure, fatal arrhythmias, embolic events (such as stroke) and infective endocarditis,^17,18^ with the majority of these deaths occurring in low- and middle-income countries (LMICs).^10^

No treatment has been shown to alter the progression of RF to RHD^19^ and therefore appropriate antibiotic treatment within 9 d of onset of GAS pharyngitis is needed to prevent RF.^20^ For this to be successful, GAS must be completely eradicated from the pharynx.^21^ A Cochrane review on antibiotics for sore throat (ST) found that antibiotics reduced RF by more than two-thirds within 1 month (risk ratio 0.27 [95% confidence interval {CI} 0.12 to 0.60]).^22^

However, the risk of RF is not linked to the severity of ST^23^ and currently there is little guidance to predict which children with sore throat are at greatest risk of RF. In some countries with high incidence of RF all children presenting with ST may be treated with an antibiotic,^24^ contributing to the ever-increasing antibiotic-resistance crisis.^25,26^ In the UK, the FeverPAIN and Centor scores are recommended to guide antibiotic prescribeng.^27^ However, these scores cannot predict all complications or be relied upon for a precise diagnosis.^28^ The risk–benefit of using such a score could therefore be different in LMICs.

This systematic review aims to identify predictive factors for RF development in patients presenting with a ST, which could form the basis of a clinical prediction tool to inform, and subsequently decrease, antibiotic prescribing.

## Methods

A review protocol was registered on PROSPERO (registration CRD42019157174). Electronic searches were conducted in MEDLINE, Embase, Cumulative Index to Nursing and Allied Health Literature and Open Grey from inception to September 2019 (see the Supplementary Materials). No language or date restrictions were applied. Experts in the field were contacted for suggestions of any relevant studies. Our search results were checked to ensure these studies were captured by our search terms. Forward and backward citation searching was carried out.

### Inclusion criteria

We included studies that followed up patients presenting with ST (including those with tonsillitis and GAS pharyngitis) and documented which of them developed RF or RHD.

We searched for predictors or risk factors for RF/RHD development (signs, symptoms and sociodemographic factors) after ST, excluding those that cannot easily be detected in a low-income setting (e.g. specific genes or molecular markers).

We included primary cohort, case–control, clinical trials and cross-sectional studies, but excluded animal studies, case series, case studies, case reports, practice guidelines, cost-effectiveness analyses and systematic reviews.

### Study selection

Title, abstract and full-text screening were carried out by two independent reviewers in Rayyan,^29^ with a third reviewer resolving any disagreements.

### Data collection

A data extraction spreadsheet was developed in Excel (Microsoft, Redmond, WA, USA) and piloted with three randomly selected studies. One reviewer extracted data from all the studies, with another reviewer checking the data extraction prior to analyses to ensure accuracy.

### Study quality appraisal and risk of bias

The quality of the case–control studies was assessed using the Newcastle-Ottawa Quality Assessment Scale.^30^ The quality of cross-sectional studies was assessed using the AXIS tool.^31^ The quality of controlled trials was assessed using the Cochrane Risk of Bias 2 tool.^32^ The quality of each study was assessed independently by two reviewers.

### Data synthesis

Meta-analysis was performed using RevMan.^33^ Odds ratios (ORs) were used for the analysis, calculated from the observed number of events reported and totals. Substantial heterogeneity (I^2^ statistic^34^ >50%) between studies was anticipated, so meta-analysis was performed using inverse variance and a random effects model.

In some studies, data for certain factors in one or more patients was missing. These patients were excluded from meta-analysis of the corresponding factor. Studies were only included in forest plots if the corresponding risk factor was not an inclusion/exclusion criterion in that study and the presence of the risk factor was reported in both ST and RF patients.

Where meta-analysis was not possible, due to differences in reporting and/or different factors measured across the studies, the results were described narratively.

## Results

**Figure 1.**
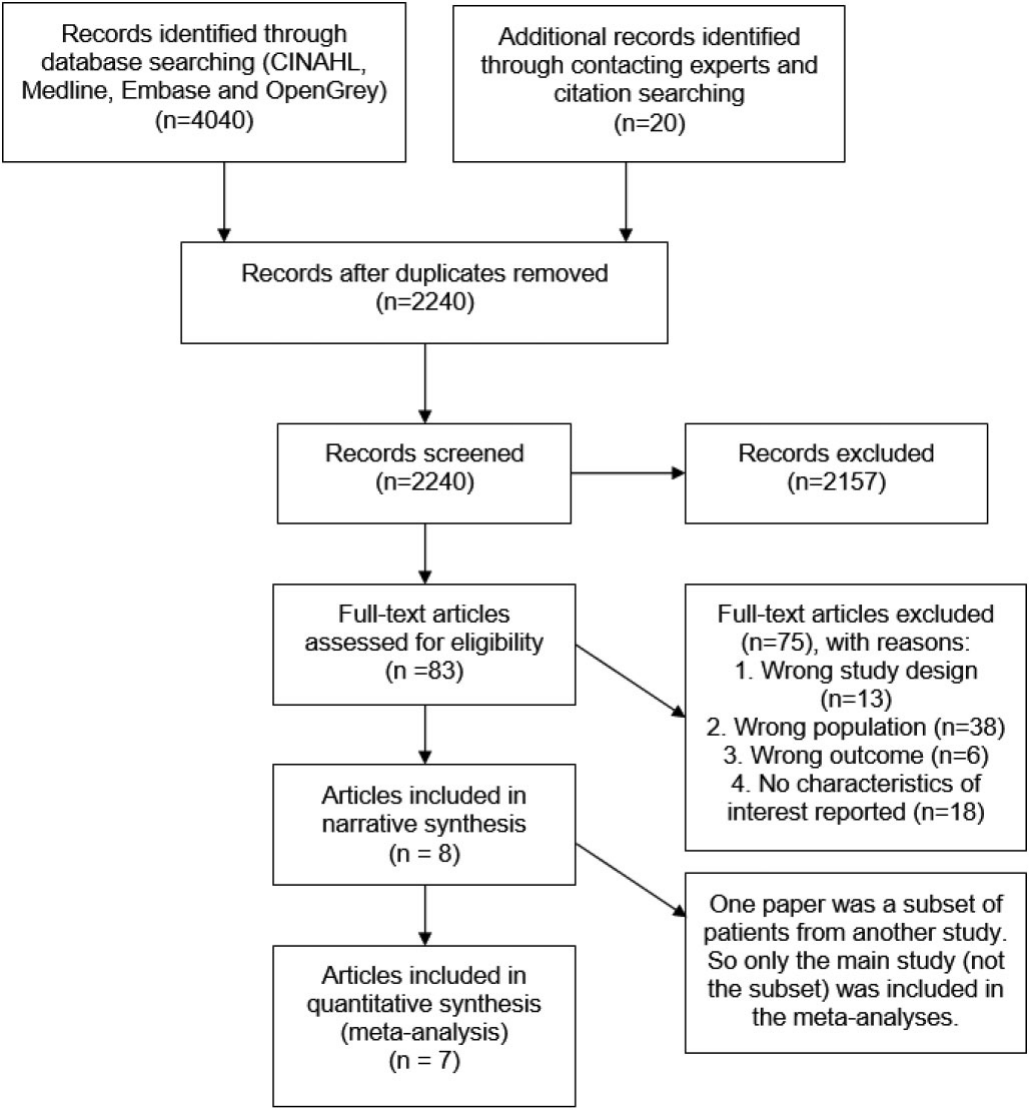
PRISMA flowchart.

Database searching identified 4040 articles (Figure 1) and 1820 duplicates were removed, leaving 2220 articles for title and abstract screening, of which 63 were selected for full-text screening. A further 20 articles were identified through forward and backward citation searching and contacting experts in the field, so a total of 83 articles underwent full-text screening. A total of 75 articles were excluded during full-text screening, most commonly because they included asymptomatic GAS carriers and/or RF patients rather than ST patients (38 articles) or because risk factors of interest, such as sociodemographic information and clinical signs and symptoms, were not reported (18 articles).

Seven studies (reported in eight articles) were included in this review with a total of 6890 participants (Table 1): three cross-sectional studies,^35–37^ one case–control study (published in two separate papers),38,39 and three randomised controlled trials (RCTs).^40–42^ All seven studies included populations of ST and RF patients, but only one^35^ also included RHD patients.

**Table 1.** Characteristics of studies included in this review

Study	Country	Study design	Total number	Age (years),^a^ mean (range)	Setting	Clinical condition	Study aims	Length of follow-up^b^
Wannamaker et al. ^40^	USA	RCT of three dosages of procaine penicillin vs no treatment	2340	20 (17–27)	US Air Force base hospital	Exudative tonsillitis or pharyngitis	Determine effect of treatment on the incidence of RF, the streptococcal carrier state and the immunologic response of the host	Planned: 3–5 weeks Selective: up to 24 weeks
Houser et al.^41^	USA	Randomised controlled trial of three treatment schedules of aureomycin vs no treatment	2044	20 (17–32)	US Air Force base hospital	Exudative tonsillitis or pharyngitis	Determine effect of treatment on the incidence of RF	Planned: 3–5 weeksSelective: up to 15 weeks
Catanzaro et al. ^42^	USA	RCT of three treatment schedules of oxytetracycline vs no treatment	986	20 (17–27)	us air force base hospital	Exudative tonsillitis or pharyngitis with a throat swab positive for streptococcus prior to treatment	Determine effect of treatment on the incidence of RF	Planned: 3–5 weeksSelective: up to 29 weeks
Negus^37^	Western Fiji	Cross-sectional	945 (180 RF, 765^c^ acute tonsillitis)	3–16 for acute tonsillitis; any age for RF	Hospital and Indian primary schools	Diagnosed RF patients and children with acute tonsillitis	To investigate the sex inequality of RF in Indians living in Fiji with particular reference to environmental factors	N/A
Tewodros et al. ^35^	Ethiopia	Cross-sectional	211^d^ (143 tonsillitis, 24 RF, 44 RHD)	8 (3–14); tonsillitis: 6.9 (3–8); RF: 9.5 (4–14); RHD: 10.4 (5–14)	Children's Hospital	Diagnosed tonsillitis, RF or RHD	Determine the prevalence of pharyngeal beta-haemolytic streptococci	N/A
Zaman et al.^38,39^	Bangladesh	Case–control study	164 (60 cases, 104 controls)	11.4 (5–20)	Patients from a national RF referral centre	Patients with or without RF, most of which had antecedent pharyngitis	Assess the association between nutritional factors and RF	N/A
			88 (44 cases and 44 age- and sex-matched controls)	12.5 (5–20)		Subset of patients from part A cohort in whom fasting convalescent blood samples were taken	Assess the association between serum albumin concentration and body iron stores and RF	
Omurzakova et al^36^	Kyrgyzstan	Cross-sectional	200	11 (3–17)	Children's Hospital	Tonsillitis or pharyngitis patients, 51 of whom had RF	Determine streptococcal carriage rate while comparing two methods of GAS detection, and the susceptibility of discovered GAS to different groups of antibiotics	N/A

a In the three studies in hospitalised airmen it was only possible to calculate the age range and the mean for the patients with RF, not the overall population.^40–42^

b In the three studies in hospitalised airmen, the authors had planned to follow up patients after 3–5 weeks, but they also reported RF occurrences up to 29 weeks after the start of the study (presumably patients who had been included in the study that happened to re-present to the clinic with RF).^40–42^

c This study included 3369 children with upper respiratory tract infections, but not all of these patients fit the inclusion criteria for the review (e.g. those with bronchitis). Therefore we only included the 765 patients with acute tonsillitis that we were certain fit our inclusion criteria.^37^

d This study included a total of 816 participants, but only 211 of them fit the inclusion criteria for this review.^35^

The RCTs, comparing different treatment schedules of antibiotics with no treatment for exudative tonsillitis or pharyngitis, were conducted on airmen from a US Air Force base in the 1950s.^40–42^ These three studies had much larger sample sizes, ranging from 986 participants to 2340, than the other studies included in this review, with sample sizes ranging from 88 to 945. The cross-sectional studies were based in hospitals in Ethiopia, Fiji and Kyrgyzstan.^35–37^ The case–control study recruited patients from a national RF referral centre in Bangladesh.^38,39^

All but one^36^ of the studies diagnosed RF using the Jones or Revised Jones Criteria—the gold standard for RF diagnosis.^43^

### Quality assessment results

Overall, one study (two articles) was judged to be of high quality, five of moderate quality and one of low quality.

All three RCTs^40–42^ were classified as having ‘some concerns’ (Table 2) because the randomisation process (via Air Force serial number) was unlikely to allow for allocation concealment, producing a risk of selection bias. Follow-up was achieved for 80–92% of participants, but this falls below the threshold of 95% and there was no information given about participants lost to follow-up. There was no statistical analysis plan. Incidental data were reported for patients who presented with RF after the study period ended, making the standard/quality of evidence questionable.

**Table 2.** Quality assessment of RCTs using the Cochrane RoB 2 tool^40–42^

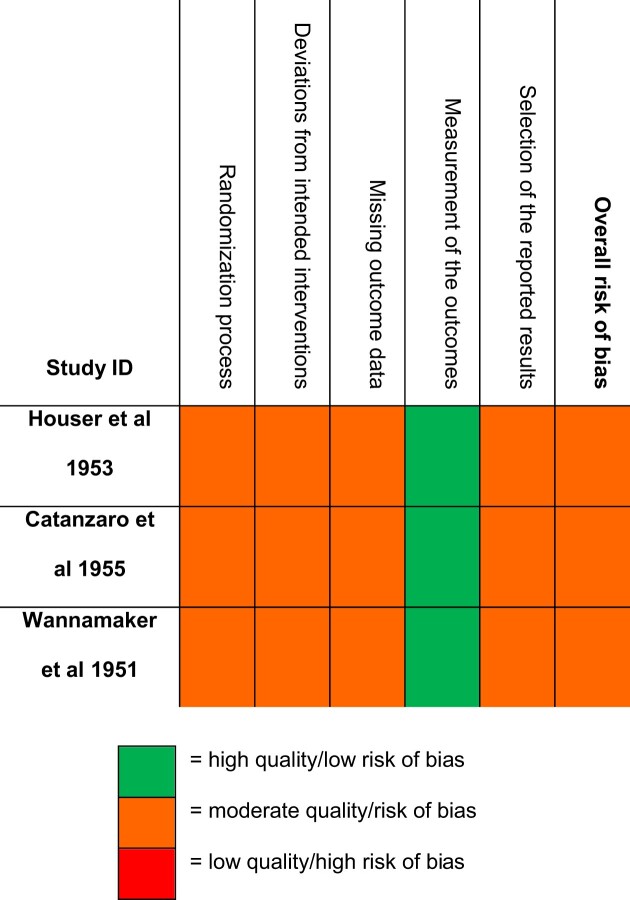

Two cross-sectional studies^35,36^ were of moderate quality and one was of low quality^37^ (Table 3); these were conducted in national referral hospitals, so the population is not representative of patients seen in primary care. The study in Kyrgyzstan^36^ did not clearly explain how patients were selected and did not explain how RF was defined. In the Ethiopian study,^35^ the RF and RHD patients were not all derived from a population of patients presenting with ST. In the Fijian paper,^37^ the aims and study design were not described clearly and the population was not ideal, with ST patients being children (ages 3–16 y) and RF patients were an entirely separate population with ages to 35 y. None presented a sample size calculation.

**Table 3.** Quality assessment results of cross-sectional studies using the AXIS tool^35–37^

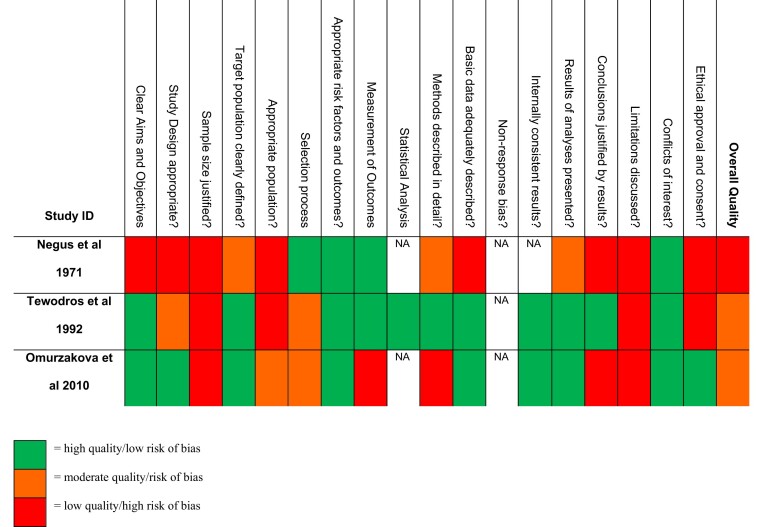

The case–control study^38,39^ was of high overall quality (Table 4).

**Table 4.** Quality assessment results of case–control study using the Newcastle-Ottawa Scale^38,39^

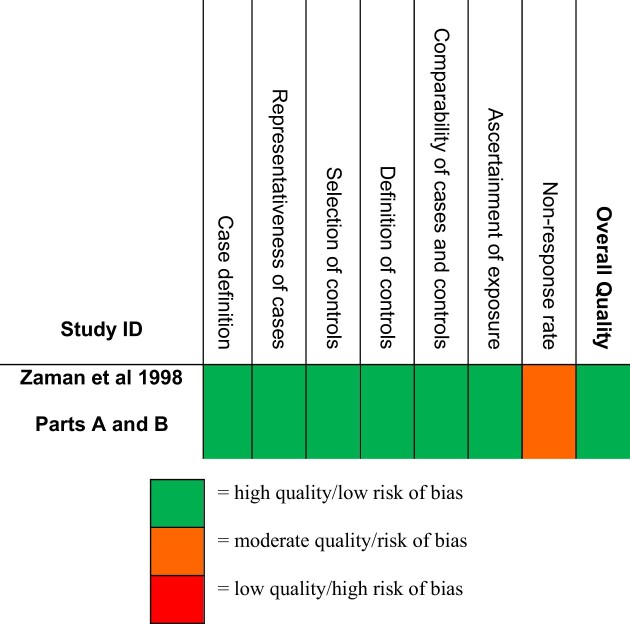

### Factors that predict RF

Risk factors that predict the development of RF after an ST are a positive swab for GAS (Figure 2.1; OR 1.74 [95% CI 1.13 to 2.69], p=0.01); a previous history of RF (Figure 2.2; OR 13.22 [95% CI 4.86 to 35.93], p<0.000 01); and the presence of a cardiac murmur upon presentation (Figure 2.3; OR 3.55 [95% CI 1.81 to 6.94], p=0.0002). The articles included in this meta-analysis were the three studies in US airmen,^40–42^ in which all participants were adults.

**Figure 2.**
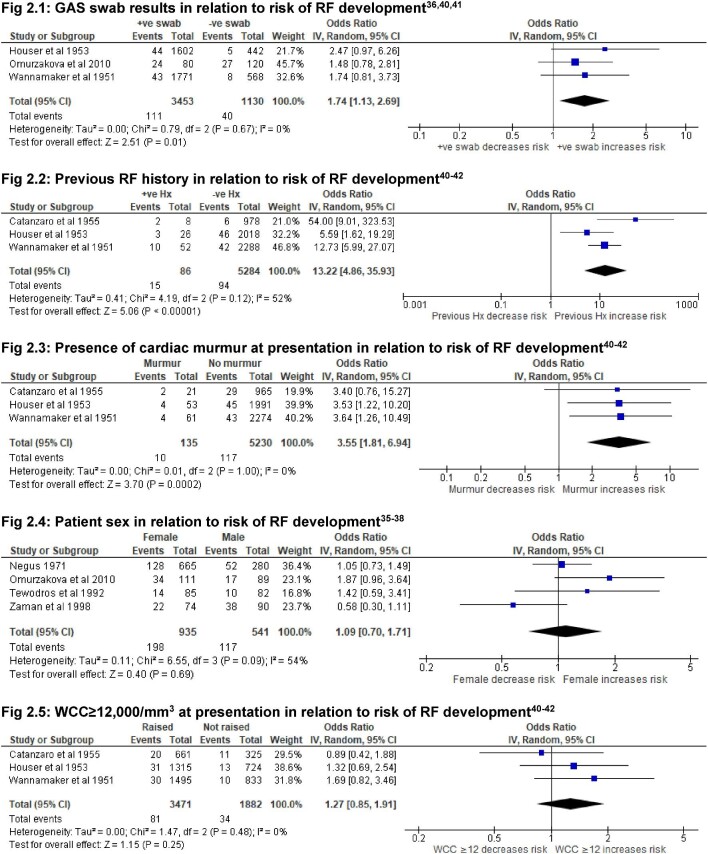
Forest plots of risk factors of RF development.

One study^35^ suggests that no access to running water is a strong and highly significant predictor of both RF development after ST (OR 12.30 [95% CI 4.66 to 32.49], p<0.000 01) and RHD development after ST (OR 4.26 [95% CI 1.98 to 9.15], p=0.0002). The case–control study^38^ demonstrated that the risk of RF increased in malnourished children (mid-upper arm circumference <80% for age; OR 2.40 [95% CI 1.04 to 5.77]) and in those with low dietary intake of eggs (OR 2.29 [95% CI 1.01 to 5.27]), even after adjusting for multiple sociodemographic confounders. It was also reported that more parental schooling in years (p<0.0001) and higher log income (p=0.002) were protective against RF development.

### Factors that do not predict RF/RHD

Sex is not a significant factor for predicting RF development after ST (Figure 2.4; OR 1.09 [95% CI 0.70 to 1.71], p=0.69). As a sensitivity analysis we excluded the article by Negus^37^ due to the high risk of bias. The heterogeneity increased slightly to 68% and the OR was 1.14 (95% CI 0.53 to 2.42), but this did not alter the inference that there is no difference between men and women. One study also included 44 RHD patients.^35^ The comparison between ST and RHD populations showed that sex was not significant for predicting RHD after ST (OR 1.01 [95% CI 0.52 to 1.99]).

### Factors with insufficient data

The meta-analysis suggests that an elevated white cell count (WCC; ≥12 000/mm^3^)^44^ upon presentation possibly increases the risk of RF development after ST, but the CI does not exclude no effect (Figure 2.5; OR 1.27 [95% CI 0.85 to 1.91], p=0.25). This may represent a lack of power in the studies included in this meta-analysis and a larger, high-quality study may be able to define the risk of an elevated WCC in the development of RF.

One study^42^ reported a family history of RF and suggests that it may predispose to RF development, but this was not statistically significant (OR 2.16 [95% CI 0.80 to 5.79], p=0.13).

Differences in the reporting of age across articles meant that meta-analysis was not possible.

#### Social factors

Data on race or ethnicity was only reported in two studies. One categorised participants as ‘Russian’ or ‘Kyrgyz’, but too few Russian participants were identified to make any meaningful comparisons between the two groups.^36^ The other reported whether RF patients were Fijian or Indian, but did not report this in the ST population, meaning the two groups could not be compared.^37^

One study^35^ reported that most of the participants with RF (70.8%) lived in crowded conditions and found crowding to be significant in univariate analysis (p=0.005), but data on crowding in the patients without RF were not reported.

#### Clinical factors

We searched for data on a previous history of recurrent tonsillitis. Two studies reported rates of tonsillectomy in the whole study population, which could be indicative of a positive previous history of recurrent tonsillitis, but this was not reported in those who then developed RF, so no comparisons could be made.^40,41^ One study selected participants from a cohort of ‘chronic tonsillopharyngitis patients’, defined as children with a history of persistent tonsillitis at least twice in a year, meaning all the participants had a history of recurring tonsillitis.^36^

One study reported whether ST patients had tonsillar exudates, but did not specify the number of cases or report this factor in the RF or RHD populations.^35^ All the US airmen had exudative tonsillitis or pharyngitis.^40–42^ Three studies (four articles) did not mention purulence/exudate.^36–39^

Maximum temperature and erythrocyte sedimentation rate were reported in the three RCTs for RF patients, but not for ST patients without RF, so no comparisons could be made between the groups.^40–42^

We searched for data on other clinical signs and symptoms, including tachycardia, tachypnoea, FeverPAIN score, Centor score, C-reactive protein level, pre-existing heart disease and any symptoms suggesting viral infection (e.g. cough or coryza), but these were not reported in any of the included studies (Table 5).

**Table 5.** Risk factors sought vs risk factors found in the included studies^35–42^

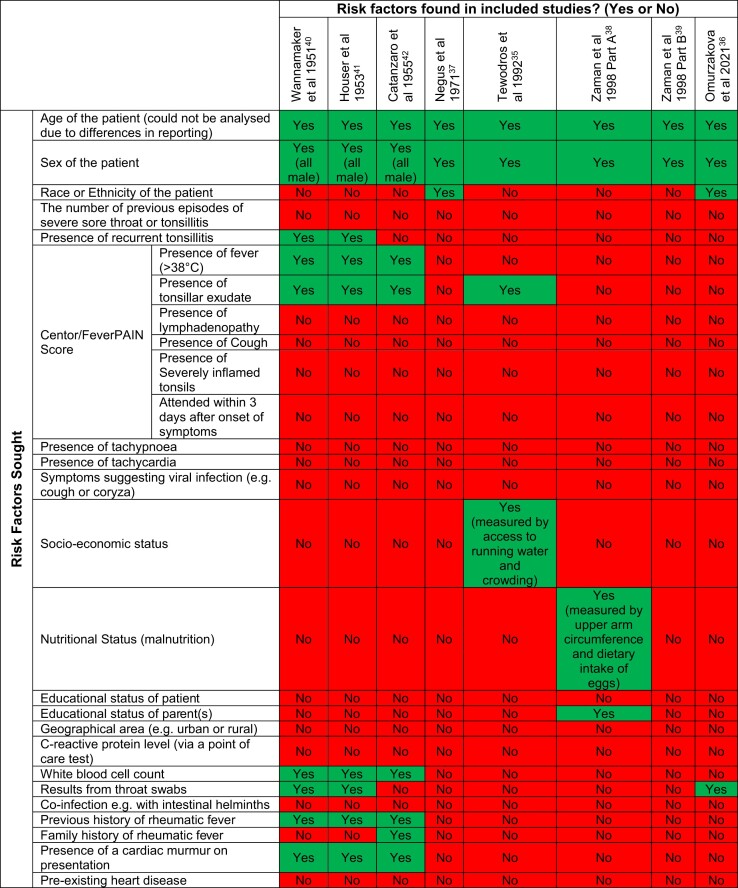

## Discussion

### Summary of findings and comparison with existing literature

This study has demonstrated that there have been no recent good-quality studies on risk factors predicting the risk of RF or RHD in patients with ST. There have been no studies to determine whether scores such as FeverPAIN, developed in high-income countries, are applicable in LMICs and can safely detect which patients with ST do not need antibiotics.

The limited evidence we found is mainly based on trials in adult men in the 1950s.^40–42^ These suggest that a positive GAS swab increased the risk of RF about 2-fold. A previous history of RF increased the risk of subsequent episodes of RF 10-fold. The presence of a cardiac murmur at presentation increased the risk of subsequent RF about 3.5-fold.

The studies that identified cardiac murmurs as a risk factor were all in adults and predated the use of echocardiography as part of the diagnosis of RF. Cardiac murmurs in adults are almost always associated with a disease^45^ and suggest underlying damage to cardiac valvular tissue.^46^ However, many febrile children have a non-pathological heart murmur,^47^ so this may not predict RF development in children. Furthermore, auscultation for a pathological murmur has been the traditional approach for RHD screening, but it is not as effective as the current gold standard of echocardiography.^48,49^ Using echocardiography instead, 10 times more cases of RHD could be detected,^50,51^ which may further increase the significance of this factor.

Long-term prophylactic antibiotics are commonly used in children with RHD to stop worsening of the heart disease with subsequent episodes.^11^ Despite this, we could not identify any articles that discussed previous heart disease as a risk factor.

Our results support the suggestion of the current literature that RF is equally common in both males and females.^52,53^ Of the seven studies included in this review, only one looked at a family history of RF as a risk factor and found it was not significant. A recent high-quality review^54^ states that the ‘risk of RF in an individual with a family history of RHD is nearly fivefold higher than that in an individual with no family history of RHD’. This suggests that in future research the factor that should be assessed in ST patients for its significance in RF development is a family history of RHD rather than RF.

Crowding and low socio-economic status are associated with an increased risk of GAS infection, RF and RHD.^55^ Factors such as crowding, malnutrition, low household income and no access to tap water in the home are used as a proxy for structural poverty and low socio-economic state. Each one of these proxy values may be measured in different ways. For example, one study measured crowding using ‘number of siblings’ and ‘number of rooms’,^35^ but another used ‘family size’ and ‘number of persons sharing a bedroom with the subject’.^38^ Future research should measure poverty/socio-economic state using standard tools to allow for comparisons between results, such as the global Multidimensional Poverty Index.^56^

### Strengths and limitations

There are currently no other published reviews comparing ST patients who developed RF with ST patients who did not develop RF. Other articles that look at characteristics of RF patients alone, or compared with healthy controls, may be identifying risk factors for GAS pharyngitis rather than RF.

The review adhered to the methods of the Preferred Reporting Items for Systematic Reviews and Meta-Analyses^57^ guidelines, the literature search was thorough, no language restrictions were applied and translations were acquired for all full-text foreign language articles.

Double screening, data extraction and quality assessment were carried out by two independent reviewers to minimise bias, with a third acting as arbiter to resolve any disagreements. Only one low-quality study was included in this review and a sensitivity analysis was performed on the meta-analysis that included this study, increasing the certainty of the results.

Three of the studies included in the review were conducted in the same location, using similar methods, within the same decade.^40–42^ This makes it very sensible to compare the results of these articles and reduces the heterogeneity between the studies.

The current population at high risk of RF are those living in LMICs and indigenous communities.^13,14^ Four of the studies (five articles) were carried out in LMICs, allowing for the results of these studies to be easily transferrable.^35–39^

We were unable to adhere to the timing and effect measures, ‘RF development up to eight weeks after ST’, for the primary outcome predetermined in the protocol published on PROSPERO because the case–control and cross-sectional studies did not report timing between ST and RF, and although the RCTs did report the interval between ST and RF occurrence, they then separately listed intervening infections for each patient, making it unclear which patients to exclude. Instead, we chose to use no timing and effect measures for the primary outcome and to analyse all available data on RF and RHD after ST.

Due to a lack of published data and differences in reporting across studies, not all of the factors we had hoped to explore in this review could be meta-analysed. More research is needed to encompass more factors and identify which ones increase RF risk in ST patients.

One of the main limitations of this review is that the three RCTs^40–42^ were carried out in a cohort of airmen in the 1950s—a population that may differ from those at risk of RF today. These studies also reported incidental data on RF development after the study period had ended, reducing the overall quality of the evidence. Furthermore, the relationship between risk factors and outcomes may be partially confounded by the different antibiotics prescribed. All of the observational studies were conducted in national referral hospitals, so the populations may be different from those seen in primary care settings.

### Implications for policy and practice

The Jones Criteria for the diagnosis of RF were revised in 2015 to include separate criteria for low-risk (those that ‘come from a setting or population known to experience low rates of RF or RHD’) and moderate- to high-risk populations (those that are ‘not clearly from a low-risk population’).^58^ However, this does not identify the risk at an individual level. Guidelines defining those at high risk could be updated to include specific factors that have been found to be significant for the development of RF after ST, in particular a previous history of RF and the presence of a cardiac murmur in adults.

### Priorities for further research

There is plenty of research that compares RF patients to a healthy population or compares patients with RHD to those with RF, as evidenced by a good quality systematic review conducted in 2018^55^ that includes 91 individual studies. However, there is insufficient research looking at the risk of RF and RHD in patients presenting with STs in order to guide antibiotic prescribing in settings where RHD is still prevalent. For example, overcrowding may increase the likelihood that an individual gets an ST but may not make them more likely to develop RF once an ST sore throat has occurred.

Overall, more research needs to be conducted that follows up ST patients in LMIC primary care settings to identify those at a higher risk of developing RF or RHD. Researchers should aim to include a range of ages, ethnicities and both male and female participants. Factors that may also be explored include patients’ previous history of RF or recurrent tonsillitis; family history of RHD; clinical symptoms and signs such as cough, coryza, joint pains, fever, severe inflammation of tonsils, purulent exudate and cardiac murmur; clinical scores (such as FeverPAIN) and point-of-care tests such as C-reactive protein level and rapid diagnostic tests for malaria (in malaria-endemic countries). Existing scores such as FeverPAIN could be evaluated and if necessary adapted to predict the risk of RF and to guide antibiotic prescription for ST in LMICs. This should be as sensitive as possible, so as not to miss any potential cases of RF, but also as specific as possible in order to reduce inappropriate use of antibiotics.

## Conclusions

This review highlights an important gap in the evidence. There are no recent data on primary care populations with ST in LMICs to identify which individuals are at risk of developing RF or RHD to inform guidelines on antibiotic prescription.

This review suggests that factors significantly associated with the development of RF following ST are a positive GAS test, a previous history of RF and the presence of a cardiac murmur at presentation (in adults).

## Supplementary Material

trab156_Supplemental_File

## Data Availability

The authors confirm that the data supporting the findings of this study are available within the article [and/or] its supplementary materials.
