# Supplementary material for: Predictors of rheumatic fever in sore throat patients: a systematic review and meta-analysis
Source: Trans R Soc Trop Med Hyg. 2021 Oct 12;116(4):286–97. doi: 10.1093/trstmh/trab156 (PMC8978297; doi:10.1093/trstmh/trab156)
Supplement: trab156_Supplemental_File [file trab156_supplemental_file.docx]

Supplementary Materials

# Search Strategy Example

*Searches completed and imported to EndNote on 24/09/2019*

**Database: Ovid MEDLINE(R) and Epub Ahead of Print, In-Process & Other Non-Indexed Citations, Daily and Versions(R) <1946 to September 11, 2019>**

Search Strategy:

1. Prognosis/
2. risk factors/
3. (predict* or caus* or develop*).mp.
4. risk* or factor*.mp.
5. (anticipat* or compar*).mp.
6. forecast*.mp.
7. outcome*.mp.
8. prognos*.mp.
9. (aetiolog* or etiolog*).mp.
10. exp Pharyngitis/
11. pharyngitis.mp.
12. tonsillitis.mp.
13. nasopharyngitis.mp.
14. (sore adj3 throat*).mp.
15. (throat* adj3 inflam*).mp.
16. (tonsil* adj3 inflam*).mp.
17. (pharyn* adj3 inflam*).mp.
18. (nasopharyn* adj3 inflam*).mp.
19. (throat* adj3 infect*).mp.
20. (tonsil* adj3 infect*).mp.
21. (pharyn* adj3 infect*).mp.
22. (nasopharyn* adj3 infect*).mp.
23. (strep* adj3 (group A or beta haemolytic or B haemolytic or beta hemolytic or B hemolytic or pyogenes)).mp.
24. gabhs adj3 (pharyn* or throat or tonsil*).mp.
25. lancefield *group A adj3 (pharyn* or throat or tonsil* or strep*).mp.
26. exp Rheumatic Fever/
27. rheumatic fever.mp.
28. (rheumatic adj5 heart).mp.
29. (rheumatic adj5 card*).mp.
30. (rheumatic adj5 aort*).mp.
31. (rheumatic adj5 mitral).mp.
32. (rheumatic adj5 valv*).mp.
33. ((ARF not respiratory failure) and (ARF not renal failure)).mp.
34. RHD.mp.
35. 1 or 2 or 3 or 4 or 5 or 6 or 7 or 8 or 9
36. 10 or 11 or 12 or 13 or 14 or 15 or 16 or 17 or 18 or 19 or 20 or 21 or 22 or 23 or 24 or 25
37. (26 or 27 or 28 or 29 or 30 or 31 or 32 or 33 or 34) not non-rheumat*.mp.
38. 35 and 36 and 37
39. 38 not ((animals/ or animal*.mp.) not (animals/ and humans/)) **(1396)**

# Table of Studies Excluded in Full Text Screening with Reasons

| **Study ID** | **Study Title** | **Exclusion Reason** |
| --- | --- | --- |
| Adam et al 2000 | Short-course antibiotic treatment of 4782 culture-proven cases of group A streptococcal tonsillopharyngitis and incidence of post-streptococcal sequelae | No characteristics provided for those who developed rheumatic fever (18) |
| Morris et al 1956 | Prevention of rheumatic fever by treatment of previous streptococcal infections: Effect of sulfadiazine |  |
| Denny et al 1950 | Prevention of rheumatic fever: Treatment of the preceding streptococcal infection |  |
| Davies et al 1973 | Problems in the primary prevention of rheumatic fever: experience from Jerusalem |  |
| Denny et al 1949 | An effective method for the prevention of rheumatic fever after the development of a streptococcal infection |  |
| Gharagozloo et al 1972 | Streptococcal infection, rheumatic fever and rheumatic heart disease among 500 Jewish school children in Teheran |  |
| Frankish et al 1978 | Rheumatic fever and streptococci: the Wairoa College study |  |
| Shiokawa 1975 | Streptococcus surveys in Ryukyu Islands, Japan |  |
| Beijing friendship hospital 1982 | A survey of rheumatic fever and related diseases among rural adolescents following epidemic streptococcal infection of upper respiratory tract. [Chinese] |  |
| Niculescu et al 1954 | Post-tonsillitis period. [Romanian] |  |
| Hahn et al 1951 | Effect of cortisone on acute streptococcal infections and post-streptococcal complications |  |
| Rammelkamp et al 1952 | Epidemiology and prevention of rheumatic fever |  |
| Siegel et al 1961 | Controlled Studies of Streptococcal Pharyngitis in a Pediatric Population — Factors Related to the Attack Rate of Rheumatic Fever |  |
| Rammelkamp et al 1951 | Prevention of rheumatic fever |  |
| Spagnuolo et al 1971 | Risk of rheumatic fever recurrences after streptococcal infections. prospective study of clinical and social factors |  |
| Brink et al 1951 | Effect of penicillin and aureomycin on the natural course of streptococcal tonsillitis and pharyngitis |  |
| Catanzaro et al 1954 | The role of streptococcus in the pathogenesis of rheumatic fever |  |
| Denny et al 1953 | Comparative effects of penicillin, aureomycin and terramycin on streptococcal tonsillitis and pharyngitis |  |
| Coburn et al 1932 | Studies on the Relationship of Streptococcus Hemolyticus to the Rheumatic Process : I. Observations on the Ecology of Hemolytic Streptococcus in Relation to the Epidemiology of Rheumatic Fever | Study design does not meet inclusion criteria (13) |
| Franco et al 1974 | Rheumatic fever in Medellin. [Spanish] |  |
| Hoyos 1964 | Epidemiology of rheumatic fever. [French] |  |
| Kuneshka et al 2013 | The epidemiological data of rheumatic heart disease in time distance. |  |
| Patten 1981 | Rheumatic fever in the West Kimberley |  |
| Vaisman 1959 | Acute tonsillitis & rheumatic fever. [Spanish] |  |
| Teodorovici et al 1977 | Evaluation of the risk of late nonsuppurative complications of infections caused by group A beta-hemolytic Streptococcus in school communities [Romanian] |  |
| Kamberg et al 1959 | Therapy of acute streptococcal tonsillitis in prevention of acute rheumatism & acute glomerulonephritis. [Dutch] |  |
| Shaughnessy 2000 | Is 5 days of antibiotic treatment effective for preventing consequences of strep throat? |  |
| Krasnova et al 1974 | Primary prophylaxis against rheumatic fever in children [Russian] |  |
| Sramek 1989 | Risk factors in rheumatic fever [Russian] |  |
| Coffey et al 2018 | The role of social determinants of health in the risk and prevention of Group A streptococcal infection, acute rheumatic fever and rheumatic heart disease: A systematic review. |  |
| Spinks et al 2013 | Antibiotics for sore throat |  |
| Bobylev et al 1995 | The risk factors for unfavorable outcomes in rheumatism. [Russian] | Population does not meet inclusion criteria (38) |
| Abdel-Moula et al 1998 | Prevalence of rheumatic heart disease among school children in Alexandria, Egypt: a prospective epidemiological study |  |
| Adanja et al 1988 | Socioeconomic factors in the etiology of rheumatic fever |  |
| Al-Sekait et al 1990 | Rheumatic heart disease in schoolchildren in western district, Saudi Arabia |  |
| Ismail et al 1982 | Epidemiology and prevention of acute rheumatic fever. Experience in a suburban area of Tunis. [French] |  |
| Campanale et al 2017 | Prevalence of Rheumatic Heart Disease in North Madagascar: An echocardiographic screening in young and adult populations |  |
| Chand 1963 | Rheumatic fever and rheumatic heart disease in Simla hills: Epidemiological aspects |  |
| Gurney et al 2016 | Estimating the risk of acute rheumatic fever in New Zealand by age, ethnicity and deprivation |  |
| Kogan et al 1969 | Long-term prevention of rheumatism [Russian] |  |
| Ngaide et al 2015 | Prevalence of rheumatic heart disease in Senegalese school children: A clinical and echocardiographic screening |  |
| Noonan et al 2013 | A national prospective surveillance study of acute rheumatic fever in Australian children |  |
| Omurzakova et al 2018 | Epidemiological Study of Rheumatic Heart Disease In Kyrgyzstan: Current State and Future Prospects |  |
| Omurzakova et al 2012 | Features of rheumatic fever in adult patients in modern Kyrgyzstan |  |
| Onan et al 2016 | Potential role of Vitamin D in pathogenesis of acute rheumatic fever |  |
| Poppi et al 1953 | Epidemiology of rheumatic fever in a rural district in Italy (with particular reference to some environmental factors) |  |
| Tsyganova et al 1980 | Incidence of rheumatism among the Taimyr population and the role of environmental factors. [Russian] |  |
| Agarwal et al 1995 | Rheumatic heart disease in India |  |
| Dobson et al 2012 | Environmental factors and rheumatic heart disease in Fiji |  |
| Gray et al 1952 | A long-term survey of rheumatic and non-rheumatic families; with particular reference to environment and heredity |  |
| Hewitt et al 1952 | Some epidemiological aspects of acute rheumatism |  |
| Kurahara et al 2006 | Ethnic differences for developing rheumatic fever in a low-income group living in Hawaii |  |
| Longo-Mbenza et al 1998 | Survey of rheumatic heart disease in school children of Kinshasa town |  |
| Okello et al 2012 | Socioeconomic and environmental risk factors among rheumatic heart disease patients in Uganda |  |
| Quinn et al 1948 | Rheumatic heart disease and crowding; a survey of rural and urban Connecticut school children |  |
| Vlanjinac et al 1991 | Influence of socio-economic and other factors on rheumatic fever occurrence |  |
| Odemis et al 2006 | Assessment of cardiac function and rheumatic heart disease in children with adenotonsillar hypertrophy |  |
| Zih 1967 | The abo blood groups in rheumatic fever and rheumatic carditis |  |
| Cuoco et al 2015 | The heart in rheumatic fever: Frequency and severity in our series |  |
| Rodrigues et al 2018 | Morbimortality of rheumatic fever and rheumatic valvulopathy in the period 2008 to 2017 in the state of Para |  |
| Omurzakova et al 2015 | Kyrgyz-Japanese international scientific and practical alpine expedition in the high valley of Kyrgyz Republic |  |
| Omurzakova et al 2014 | Study about prevalence of group A streptococcus and clinical-functional assessment of rheumatic fever in Kyrgyz Republic |  |
| Omurzakova et al 2016 | Study on prevalence and clinical assessment of rheumatic fever in the Kyrgyz republic |  |
| Omurzakova et al 2014 | Prevalence of group A b-hemolytic streptococcus and clinical-functional peculiarities assessment of rheumatic fever in the Kyrgyz Republic |  |
| Beaudoin et al 2014 | Assessing the burden of pediatric acute rheumatic fever and rheumatic heart disease-American Samoa, 2011-2012 |  |
| Marjanovic 1975 | Rheumatic fever in the Krusevac area during the period from 1958 to 1972. Epidemiological observations (Serbocroatian). [Serbian] |  |
| Powell et al 1981 | Acute rheumatic fever in Mississippi: a survey of hospitalized cases, 1964 to 1973. |  |
| Grave 1957 | Social and environmental factors in the aetiology of rheumatic fever |  |
| Zaman et al 1997 | Socio-economic deprivation associated with acute rheumatic fever. A hospital-based case-control study in Bangladesh. |  |
| Nicholas et al 1962 | Occurrence of groupable beta-hemolytic streptococci: study among school children in bismarck | Outcome does not meet inclusion criteria (6) |
| Baghchehsaraei et al 2010 | Study of antistreptolysin O (ASO) titer in patients suspected to rheumatic fever in Zanjan |  |
| Werthein et al 1974 | Epidemiological study on the incidence of streptococcal infection in an elementary school population. [Spanish] |  |
| Antoncecchi et al 1979 | Amoxicillin in the prevention of rheumatic fever [Italian] |  |
| Teodorovici et al 1973 | Epidemiological survey of streptococcal infections in a large school unit [Romanian] |  |
| Wiley et al 1973 | Clinical isolation of streptococcus pyogenes seasonal prevalence in Nebraska |  |
